# White matter hyperintensities across the adult lifespan: relation to age, Aβ load, and cognition

**DOI:** 10.1186/s13195-020-00669-4

**Published:** 2020-10-08

**Authors:** Antoine Garnier-Crussard, Salma Bougacha, Miranka Wirth, Claire André, Marion Delarue, Brigitte Landeau, Florence Mézenge, Elizabeth Kuhn, Julie Gonneaud, Anne Chocat, Anne Quillard, Eglantine Ferrand-Devouge, Vincent de La Sayette, Denis Vivien, Pierre Krolak-Salmon, Gaël Chételat

**Affiliations:** 1 Normandie Univ, UNICAEN, INSERM, U1237, PhIND “Physiopathology and Imaging of Neurological Disorders”, Institut Blood and Brain @ Caen-Normandie, Cyceron, 14000 Caen, France; 2 Clinical and Research Memory Center of Lyon, Lyon Institute For Elderly, Charpennes Hospital, Hospices Civils de Lyon, Lyon, France; 3 Claude Bernard University Lyon 1, Lyon, France; 4 German Center for Neurodegenerative Diseases (DZNE), Dresden, Germany; 5 Normandie Univ, UNICAEN, PSL Université, EPHE, INSERM, U1077, CHU de Caen, GIP Cyceron, NIMH, Caen, France; 6 Department of General Practice, Normandie Univ, UNIROUEN, Rouen, France; 7 Rouen University Hospital, Inserm CIC-CRB 1404, F-76 000 Rouen, France; 8 Department of Neurology, CHU de Caen, Caen, France; 9 Department of Clinical Research, CHU de Caen, Caen, France; 10 Lyon Neuroscience Research Centre, INSERM 1028, CNRS 5292, Lyon, France

**Keywords:** White matter hyperintensities, Cortical Aβ, Cognition

## Abstract

**Background:**

White matter hyperintensities (WMH) are very frequent in older adults and associated with worse cognitive performance. Little is known about the links between WMH and vascular risk factors, cortical β-amyloid (Aβ) load, and cognition in cognitively unimpaired adults across the entire lifespan, especially in young and middle-aged adults.

**Methods:**

One hundred and thirty-seven cognitively unimpaired adults from the community were enrolled (IMAP cohort). Participants underwent (i) a comprehensive neuropsychological assessment of episodic memory, processing speed, working memory, and executive functions; (ii) brain structural T1 and FLAIR MRI scans used for the automatic segmentation of total and regional (frontal, parietal, temporal, occipital, and corpus callosum) WMH; and (iii) a Florbetapir-PET scan to measure cortical Aβ. The relationships of total and regional WMH to age, vascular risk factors, cortical Aβ, and cognition were assessed within the whole sample, but also splitting the sample in two age groups (≤ or > 60 years old).

**Results:**

WMH increased with age across the adult lifespan, i.e., even in young and middle-aged adults. Systolic blood pressure, diastolic blood pressure, and glycated hemoglobin were all associated with higher WMH before, but not after, adjusting for age and the other vascular risk factors. Higher frontal, temporal, and occipital WMH were associated with greater Aβ, but this association was no longer significant when adjusting for age and vascular risk factors. Higher total and frontal WMH were associated with worse performance in executive functions, with no interactive effect of the age group. In contrast, there was a significant interaction of the age group on the link between WMH and working memory, which was significant within the subgroup of young/middle-aged adults only. Adding cortical Aβ load in the models did not alter the results, and there was no interaction between WMH and Aβ on cognition.

**Conclusion:**

WMH increased with age and were associated with worse executive functions across the adult lifespan and with worse working memory in young/middle-aged adults. Aβ load was weakly associated with WMH and did not change the relationship found between WMH and executive functions. This study argues for the clinical relevance of WMH across the adult lifespan, even in young and middle-aged adults with low WMH.

## Background

White matter hyperintensities (WMH), visible on T2-weighted MRI scans, are frequent in the elderly population, being in patients with cognitive impairment but also in cognitively unimpaired adults older than 60 years old. There is converging evidence for a relationship between increased WMH and decreased cognitive performance, mostly for executive functions, attention, and processing speed, even in older adults with normal cognition [1, 2]. In contrast, only a few studies have investigated the prevalence and regional specificity of WMH in young and middle-aged adults [3–7] or their link with cognition [7–13], and findings are contrasted. Overall, they showed a non-linear increase in WMH with age, with a steeper increase from the fifth or sixth decade [4, 7]. The midlife period has been previously described as a critical period for cerebrovascular health and regulation of vascular risk factors [14, 15] and might thus represent a relevant window for cerebrovascular prevention. Further assessments of WMH in young and middle-aged adults, and of their clinical relevance, thus seem of particular interest to improve our understanding of their impact, and eventually design and monitor prevention interventions.

The presence of cortical β-amyloid (Aβ) deposition is another relevant factor to consider in this context as it is one of the main pathological landmark of Alzheimer’s disease and it significantly increases with age, including in cognitively unimpaired individuals [16]. Recent findings even showed linear increase in amyloid PET tracer binding from 20 to 60 years old [17]. The association between WMH and cortical Aβ is still debated [18–21] with some studies suggesting that WMH and cortical Aβ are independent and additive pathological processes leading to cognitive deficits while others propose that the lesions interact and have synergistic effects on cognition [19, 22–26].

This study aims at providing further insight on total and regional WMH in cognitively unimpaired individuals covering the entire adult lifespan. We will assess the WMH relation to age, vascular risk factors, cortical Aβ, and cognition. Then, we will assess the possible interaction between WMH and cortical Aβ on cognition across the adult lifespan.

## Methods

### Participants

One hundred and thirty-seven cognitively unimpaired individuals from the IMAP study (multimodal neuroimaging of early Alzheimer’s disease) [27], aged between 19 and 85 (50 participants < 40 years old, 36 between 40 and 60 years old, and 51 > 60 years old), were included in this study. Participants were recruited from the general population through advertisement or word of mouth. They had no history or clinical evidence of major neurological or psychiatric disorder and performed in the normal range in all neuropsychological tests (including tests of episodic memory, working memory, language skills, executive functions, and visuospatial abilities). A regional review board has approved the use of human participants for this study, and consent forms from all participants were obtained. All neuropsychological, MRI, and PET assessments were performed in close temporal proximity (within 3 months). Demographic, clinical, and neuroimaging data are summarized in Table 1 (and Additional file: Table S1 for the two age groups, see below).

**Table 1:** Demographic, clinical, and neuroimaging data in the whole population

*N*	137
Age	49.99 ± 19.66 [19–85]
Sex (% male)	50% (*N* = 69)
Level of education (years)	13.11 ± 3.30 [7–20]
SBP^1^ (mmHg)	133.92 ± 19.27 [95–198]
DBP^1^ (mmHg)	78.32 ± 11.03 [54–118]
HbA1C^2^ (%)	5.46 ± 0.40 [4.50–6.80]
Episodic memory^3^	0.03 ± 0.68 [− 2.11–1.89]
Processing speed^3^	0.00 ± 0.84 [− 2.77–1.77]
Working memory^3^	0.00 ± 0.86 [− 1.60–2.58]
Executive functions^3^	0.00 ± 0.66 [− 2.66–1.41]
Total WMH (cm^3^)	2.74 ± 5.07 [0–34.90]
Frontal WMH (cm^3^)	0.91 ± 2.02 [0–14.92]
Parietal WMH (cm^3^)	0.68 ± 1.57 [0–9.57]
Temporal WMH (cm^3^)	0.33 ± 0.60 [0–3.11]
Occipital WMH (cm^3^)	0.34 ± 0.63 [0–4.65]
Callosal WMH (cm^3^)	0.33 ± 0.65 [0–4.09]
Aβ load^4^ (SUVR)	1.17 ± 0.10 [0.97–1.64]
Aβ positive participants^4^ (% ; Number)	6% (N=6)
TIV (dm^3^)	1.37 ± 0.13 [1.09–1.62]

Numbers are expressed as mean ± standard deviation; the ranges are shown in brackets, or percentage (for the sex)

*SBP* systolic blood pressure, *DBP* diastolic blood pressure, *HbA1C* glycated hemoglobin, *WMH* white matter hyperintensities, *TIV* total intracranial volume

^1^Data available for 133 participants

^2^Data available for 128 participants

^3^Data available for 136 participants

^4^Data available for 109 participants

### Cognitive assessment

The participants underwent a comprehensive neuropsychological assessment as described in details previously [28]. To obtain robust proxies of cognitive performance and minimize the issue of multiple statistical testing, individual scores were *z*-scored (using the whole sample as the reference) and averaged to compute four individual composite scores reflecting episodic memory, processing speed, working memory, and executive functions. The cognitive scores were available for 136 participants. The episodic memory score included the immediate free recalls and delayed free recall of the Free and Cued Selective Reminding Test [29] (FCSRT; available in all participants ≥ 40 years old), the memory subscore of the Mattis Dementia Rating Scale [30], and the free recalls of the “encoding, storage, retrieval” paradigm [31]. The processing speed composite score was obtained from the scores (times) at the Trail Making Test (TMT) part A, and color reading and color naming of the Stroop test [32]. The working memory composite score included performance in the forward and backward digit span [33]. The executive functions’ composite score was computed from the score at the letter verbal fluency test [32], a score of flexibility calculated on the TMT [32] (time difference between parts B and A divided by time of part A), and a score of inhibition computed from the Stroop test [32] (time difference between interference and naming tasks). Composite scores were computed in participants in which at least two corresponding individual scores were available. Note that before being averaged, *z*-scores derived from reaction times (i.e., TMT and Stroop) were reversed (multiplied by − 1) so that higher values always indicated better performance. The correlation matrix between composite scores is presented in Additional file 1 (Figure S1).

### Neuroimaging data acquisition and processing

#### Magnetic resonance imaging

MRI scans were acquired at the Cyceron Center (Caen, France) on a Philips (Eindhoven, The Netherlands) Achieva 3T scanner using a 3D fast-field echo sequence. A high-resolution T1-weighted anatomical volume was first acquired using a 3D fast-field echo sequence (3D-T1-FFE sagittal; 180 slices, no gap; slice thickness = 1 mm; field of view = 256 × 256 mm^2^; in-plane resolution = 1 × 1 mm^2^; repetition time TR = 20 ms; echo time TE = 4.6 ms; flip angle = 10). Then, a high-resolution T2-weighted FLAIR anatomical volume was collected (3D-IR sagittal; TR/TE/TI = 8000/348/2400 ms; flip angle = 90°; 90 slices, no gap; slice thickness = 2 mm; field of view = 250 × 250 mm^2^; in-plane resolution = 0.78 × 0.78 mm^2^). The T1-weighted images were segmented, spatially normalized to the Montreal Neurological Institute (MNI) space using the segmentation routine implemented in Statistical Parametric Mapping 12 (SPM12). Total intracranial volume (TIV) was estimated using SPM12.

#### Assessment of WMH

Raw FLAIR images were coregistered to their corresponding native space T1-weighted scan, and white matter lesions were segmented using the lesion prediction algorithm (LPA (Schmidt [34], Chapter 6.1)) as implemented in the Lesion Segmentation Toolbox version 2.0.15 (www.statistical-modelling.de/lst.html) for SPM (SPM12, MatLab v.2018b; MathWorks, Natick, MA), based on the calculation of a lesion probability score for each voxel. A minimum extend threshold was set to 0.01 cm^3^. Lesion probability maps were binarized by applying a threshold of 0.5 [35], and lesion masks were thereby generated. Lesion masks were visually inspected and corrected for false positives in corticospinal tracts if necessary using a specific corticospinal tract mask for each participant (corticospinal hyperintensities are common artifacts in WMH segmentations [36]). Next, Hammers and JHU white matter adult atlases [37–39] were normalized back to the native space of each individual binary lesion map to extract the mean regional WMH volumes in the frontal, temporal, parietal, and occipital lobes and in the corpus callosum. WMH volume in cubic centimeter was defined as the voxel size multiplied by the total number of voxels labeled as lesions in the cerebrum.

#### Florbetapir (F^18^-AV45) PET

Florbetapir-PET data were available for 109 participants. Florbetapir-PET scans were acquired on a Discovery RX VCT 64 PET-CT device (General Electric Healthcare) at the Cyceron Center (Caen, France) to measure cortical Aβ load. Participants underwent an intravenous injection of ~ 4 MBq/kg of Florbetapir, and scans were acquired with a resolution of 3.76 × 3.76 × 4.9 mm^3^ (field of view = 157 mm). Forty-seven planes were obtained with a voxel size of 1.95 × 1.95 × 3.27 mm^3^. A transmission scan was performed for attenuation correction before acquisition. PET images were coregistered onto the corresponding MRI and spatially normalized using the deformation parameters derived from the MRI procedure. The resulting images underwent quantitative scaling, using cerebellar gray matter as a reference to obtain standardized uptake value ratio (SUVR) images. Finally, Florbetapir SUVR images were masked to exclude non-gray matter voxels. Normalized and scaled Florbetapir-PET images were used to extract an averaged index of cortical Aβ, using a predetermined neocortical mask (including the entire gray matter, except the cerebellum, occipital and sensory motor cortices, hippocampi, amygdala, and basal nuclei) [40]. Florbetapir-PET SUVR data were mainly used as a continuous variable in the analyses; for some analyses, however, they were also dichotomized as amyloid positive versus negative using a cutoff corresponding to the 99.9th percentile of SUVR distribution among cognitively unimpaired young adults aged < 40 years (*n* = 45) [41].

### Assessment of covariates

Sociodemographic variables consisted of age, sex, and level of education. Vascular risk factors were systolic blood pressure (SBP), diastolic blood pressure (DBP), and glycated hemoglobin (HbA1C, 9 missing data). SBP and DBP (4 missing data) were averaged over 6 assessments (three consecutive measures at two different times). The correlation matrix between age and the different vascular risk factors is presented in Additional file 1 (Figure S1).

### Statistical analyses

Statistical analyses were performed using the R software version 3.5.2, 2018 (R Core Team, www.R-project.org). In models where WMH were included as dependent variables, due to the non-normal distribution of WMH, raw WMH values (+ 0.01 to avoid zeros) were log-transformed as usually performed [5, 13, 42].

To assess the relationship between log-transformed WMH (total and regional volumes), age, vascular risk factors, and cortical Aβ, regression analyses were performed using log-transformed WMH volume as the dependent variable, and age, sex, SBP, DBP, HbA1C, Aβ, and TIV as predictors. Model 1 was only adjusted for TIV, and model 2 was additionally adjusted for all significant covariates in model 1 (among age, sex, SBP, DBP, HbA1C, and Aβ). Age was treated as a continuous variable in all these analyses.

Then, we wanted to assess whether the age effect on total WMH (log-transformed and raw volumes) was present even in young and middle-aged adults. For this purpose, participants were split in two age groups: young and middle-aged adults ≤ 60 years old (*N* = 86, mean age 37.41 ± 12.85) and older adults > 60 years old (*N* = 51, mean age 71.20 ± 6.26) (details in Additional file: Table S1). First, we added the age group (as a binary variable) as an interactive term in models 1 and 2 and tested for the possible interaction of this factor. Second, we rerun all analyses (models 1 and 2) within each age group separately. Finally, for the sake of completeness, we rerun all analyses in a subgroup of young adults ≤ 40 years old (*N* = 50, mean age 27.86 ± 6.13).

Then, to assess the relationship between WMH (total and regional volumes) and cognition, multiple regression analyses were performed using each composite cognitive score as the dependent variable and raw WMH as a predictor, controlling for age, sex, level of education, and TIV (model A). We also tested for the interactive effect of the age group (as a binary variable) and, when significant, repeated the same analyses within each age group separately. The model was next rerun for each cognitive score including Aβ as a second predictor (model B) to evaluate if the WMH effects on cognition persisted when controlling for the possible effect of Aβ. Finally, a WMH by Aβ interaction term was added (model C) to assess whether these two variables had an interactive effect on cognition. In case of missing data, the participant was excluded from the corresponding analysis.

The significance level was set to *p* <  0.05. For analyses across the whole sample, Bonferroni-corrected *p* values accounting for multiple testing across the 4 cognitive domains (episodic memory, processing speed, working memory, and executive functions) were calculated.

## Results

### Relationships of WMH to age, vascular risk factors, and Aβ

**Fig. 1:**
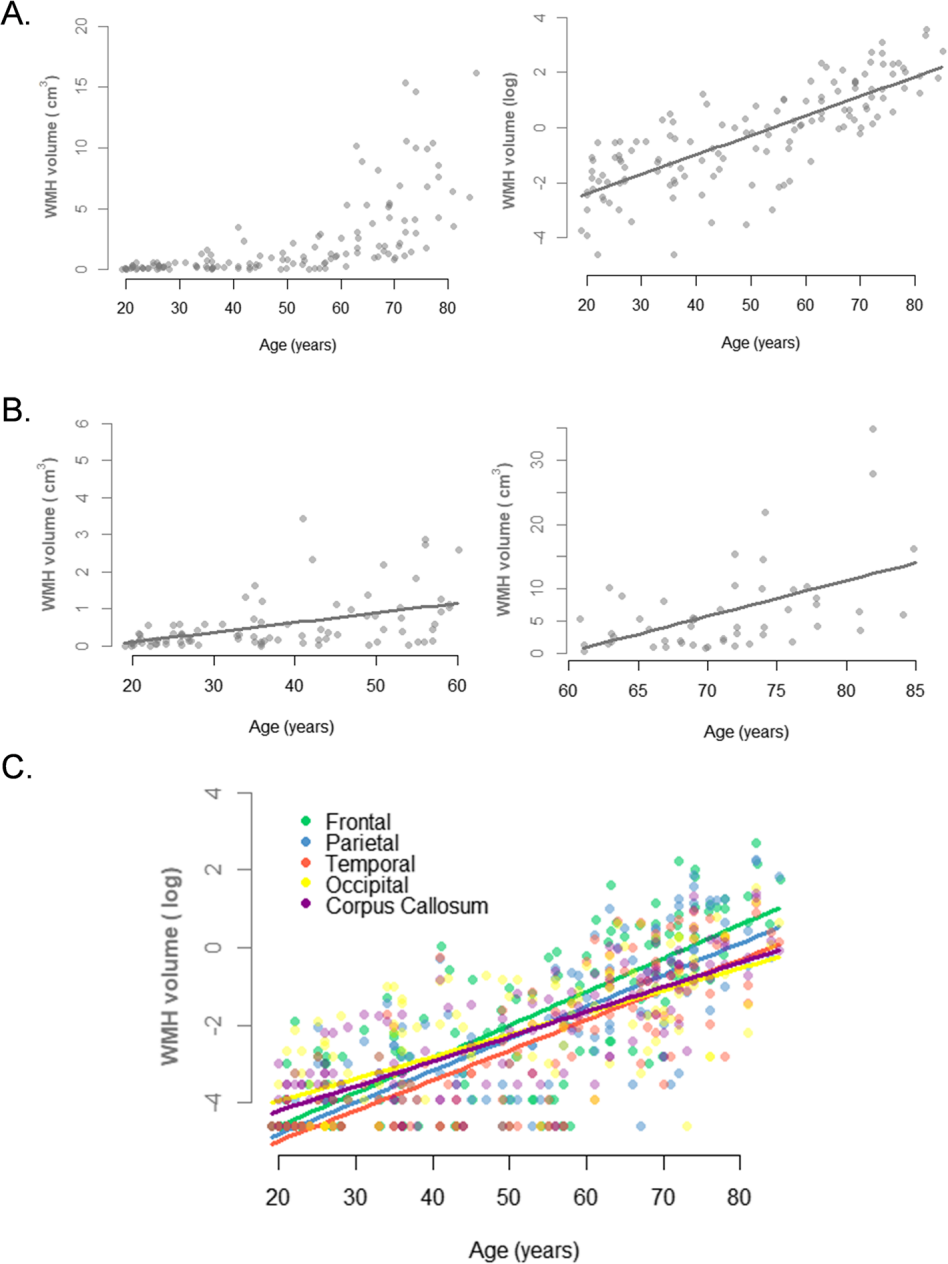
Relationship between WMH and age across the adult lifespan. **a** Total WMH as a function of age across the adult lifespan (raw volumes on the left; log-transformed volumes and linear regression curve on the right). **b** Total raw WMH as a function of age in young/middle-aged adults (on the left) and in older adults (on the right), with regression linear curve; the scale of the *y* axis is different for the two groups. **c** Regional log-transformed WMH (frontal, parietal, temporal, occipital, and corpus callosum) as a function of age across the adult lifespan

Increased total and regional log-transformed WMH were associated with increased age in the whole population, both in models 1 and 2 (Fig. 1a, c; Table 2). In addition, SBP, DBP, and HbA1C were positively associated with global and regional WMH in model 1 (only adjusted for TIV), i.e., higher WMH were associated with higher SBP, DBP, or HbA1C. These associations were no longer significant in model 2 except for SBP and parietal WMH (Table 2).

**Table 2:** Relationships between WMH, age, vascular risk factors, and Aβ

	Total WMH					Frontal WMH					Parietal WMH				
	*Model 1*		*Model 2*			*Model 1*		*Model 2*			*Model 1*		*Model 2*		
	*β*	*p*	*β*	*p*	*R*^2^	*β*	*p*	*β*	*p*	*R*^2^	*β*	*p*	*β*	*p*	*R*^2^
Age	0.80	**< 0.001**	0.73	**< 0.001**	0.66	0.81	**< 0.001**	0.79	**< 0.001**	0.69	0.80	**< 0.001**	0.77	**< 0.001**	0.69
Sex	0.00	0.99	–	–		− 0.01	0.90	–	–		0.10	0.41	–	–	
SBP	0.51	**< 0.001**	0.13	0.10		0.51	**< 0.001**	0.12	0.17		0.55	**< 0.001**	0.22	**< 0.01**	
DBP	0.41	**< 0.001**	− 0.02	0.78		0.41	**< 0.001**	− 0.05	0.56		0.40	**< 0.001**	− 0.11	0.13	
HbA1C	0.52	**< 0.001**	0.00	0.95		0.53	**< 0.001**	0.02	0.81		0.51	**< 0.001**	− 0.03	0.61	
Aβ	0.19	0.05	–	–		0.19	**0.04**	− 0.02	0.68		0.09	0.34	–	–	
	Temporal WMH					Occipital WMH					Callosal WMH				
	*Model 1*		*Model 2*			*Model 1*		*Model 2*			*Model 1*		*Model 2*		
	*β*	*p*	*β*	*p*	*R*^2^	*β*	*p*	*β*	*p*	*R*^2^	*β*	*p*	*β*	*p*	*R*^2^
Age	0.83	**< 0.001**	0.79	**< 0.001**	0.71	0.69	**< 0.001**	0.58	**< 0.001**	0.51	0.77	**< 0.001**	0.74	**< 0.001**	0.61
Sex	0.04	0.73	–	–		0.12	0.32	–	–		− 0.07	0.58	–	–	
SBP	0.48	**< 0.001**	0.07	0.38		0.47	**< 0.001**	0.20	0.07		0.51	**< 0.001**	0.14	0.11	
DBP	0.37	**< 0.001**	− 0.06	0.43		0.30	**< 0.001**	− 0.10	0.31		0.40	**< 0.001**	− 0.03	0.75	
HbA1C	0.55	**< 0.001**	0.07	0.33		0.48	**< 0.001**	0.07	0.43		0.48	**< 0.001**	− 0.04	0.58	
Aβ	0.23	**0.02**	0.00	0.97		0.20	**0.04**	0.05	0.48		0.15	0.11	–	–	

Standardized betas (*β*) and *p* values are reported from regression models where total and regional WMH (log-transformed) are regressed onto age, sex, SBP, DBP, HbA1C, and Aβ controlling for TIV (model 1) or TIV and significant covariates (model 2). Adjusted *R*^2^ values were indicated for model 2. Significant *p* values (< 0.05) are in bold

Analyses were performed with 137 participants for age and sex, 133 participants for SBP and DBP, 128 participants for HbA1C, and 109 participants for Aβ in model 1. Analyses were performed with 125 participants (for total, parietal, and callosal WMH) and 99 participants when Aβ was included (for frontal, temporal, and occipital WMH) in model 2. *SBP* systolic blood pressure, *DBP* diastolic blood pressure, *HbA1C* glycated hemoglobin, *Aβ* cortical β-amyloid, *TIV* total intracranial volume

Frontal, temporal, and occipital WMH were positively associated with Aβ in model 1, i.e., higher WMH in these regions were associated with higher Aβ load, but this association was not found in model 2 (Table 2). This result was identical when using Aβ as a binary, instead of a continuous variable.

There was no significant interaction of the age group on the relationship between age and total WMH (*p* = 0.10), indicating that the effect of age on log-transformed WMH is present and similar within the older adults and the young/middle-aged participants (Additional File: Figure S2). Increased total log-transformed WMH with age was even found in the subgroup of adults younger than 40 years old (see Additional file 1: Table S2 and Figure S3). As expected, when considering raw WMH (instead of log-transformed WMH), a significant interaction of the age group was found (*p* <  0.001), with a steeper decline in older adults than in young/middle-aged participants (Fig. 1b). Moreover, there was no interaction of the age group on the relationship between SBP, DBP, HbA1C, and total WMH, neither in model 1 nor in model 2. In contrast, we found in model 1 an interactive effect of the age group on the link between Aβ and WMH (*p* = 0.01), but this effect was no longer significant in model 2. Results within each age group are detailed in Table 3.

**Table 3:** Relationships between total WMH, age, vascular risk factors, and Aβ in the subgroup of young/middle-aged adults (A) and in the subgroup of older adults (B)

(A)	Total WMH					(B)	Total WMH				
	*Model 1*		*Model 2*				*Model 1*		*Model 2*		
	*β*	*p*	*β*	*p*	*R*^2^		*β*	*p*	*β*	*p*	*R*^2^
Age	0.47	**< 0.001**	0.35	**0.01**	0.32	Age	0.53	**< 0.001**	0.42	**0.01**	0.26
Sex	− 0.05	0.72	–	–		Sex	− 0.03	0.89	–	–	
SBP	0.24	**0.02**	0.01	0.96		SBP	0.35	**0.02**	0.10	0.51	
DBP	0.37	**< 0.001**	0.23	0.10		DBP	0.19	0.19	–	–	
HbA1C	0.20	0.06	–	–		HbA1C	0.39	**0.01**	0.20	0.17	
Aβ	0.24	**0.05**	0.09	0.39		Aβ	− 0.23	0.14	–	–	

Standardized betas (*β*) and *p* values are reported from regression models where total WMH (log-transformed) are regressed onto age, sex, SBP, DBP, HbA1C, and Aβ controlling for TIV (model 1), or for TIV and significant covariates (model 2) in young/middle-aged adults (A; *N* = 86, mean age 37.41 ± 12.85) and in older adults (B, *N* = 51, mean age 71.20 ± 6.26). Adjusted *R*^2^ were indicated for model 2. Significant *p* values (< 0.05) are in bold

For young/middle-aged adults, analyses were performed with 86 participants for age and sex, 85 participants for SBP and DBP, 80 participants for HbA1C, and 65 participants for Aβ in model 1 and with 65 participants in model 2. For older adults, analyses were performed with 51 participants for age and sex; 48 participants for SBP, DBP, and HbA1C; and 44 participants for Aβ in model 1 and with 45 participants in model 2

*SBP* systolic blood pressure, *DBP* diastolic blood pressure, *HbA1C* glycated hemoglobin, *Aβ* cortical β-amyloid, *TIV* total intracranial volume

### Associations between WMH and cognition

**Fig. 2:**
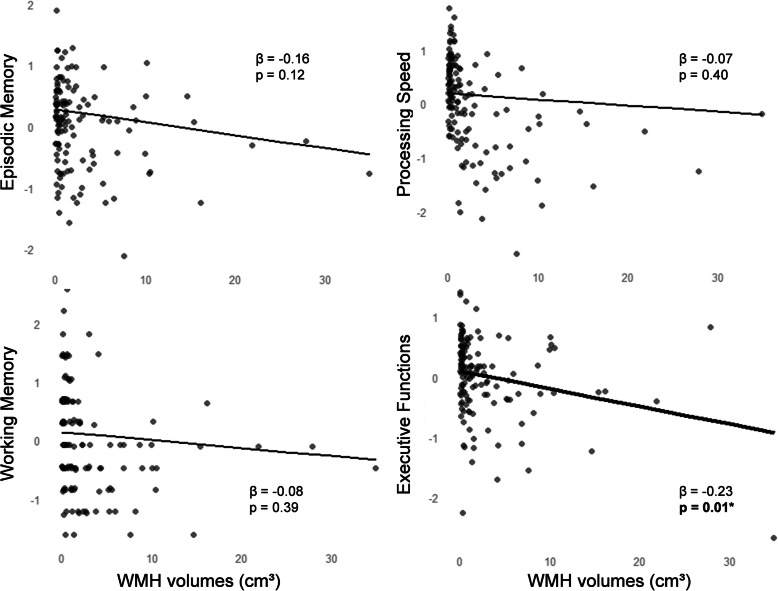
Association between total WMH and cognition. Plots represent cognitive scores (episodic memory, processing speed, working memory, and executive functions) regressed onto total WMH, controlling for age, sex, level of education, and TIV. Uncorrected *p* values < 0.05 are in bold. *p < 0.05 after applying Bonferroni correction

**Fig. 3:**
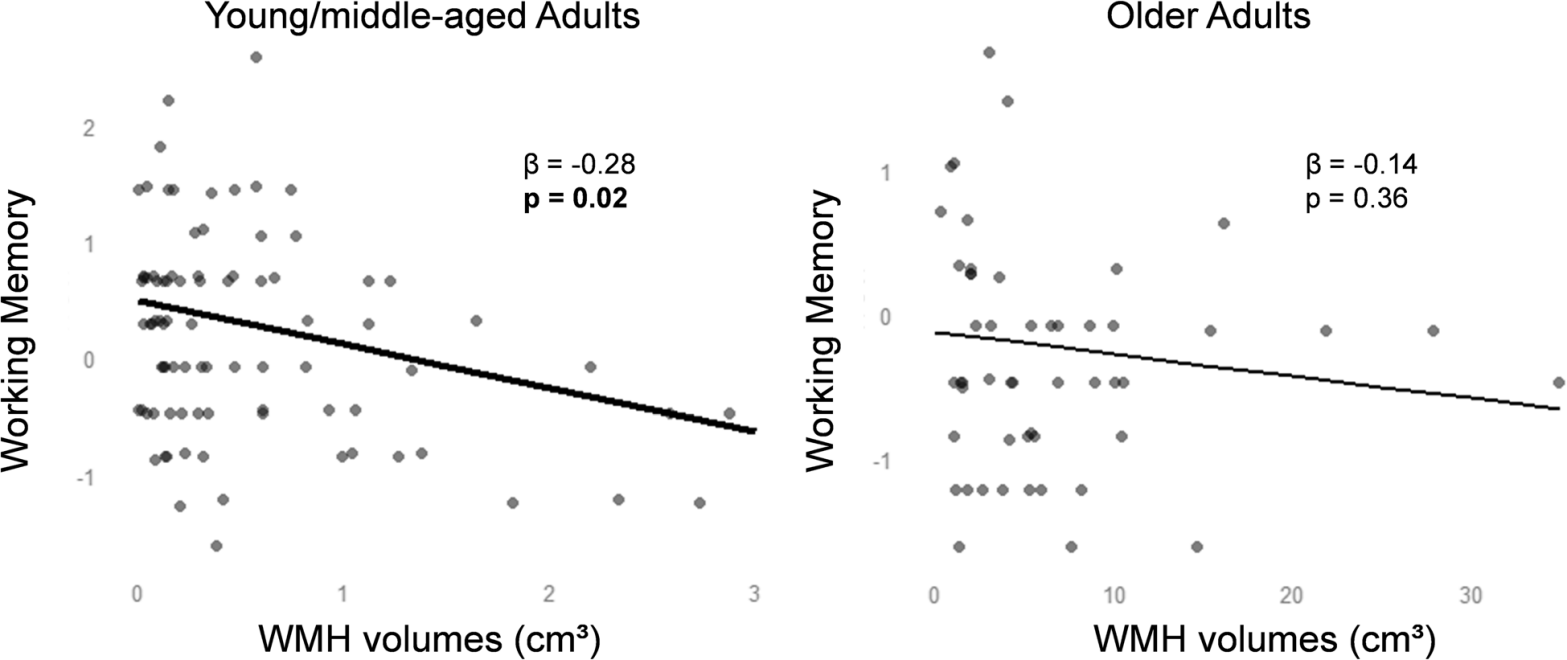
Association between total WMH and working memory in the subgroup of young/middle-aged adults and in the subgroup of older adults. Plots represent composite scores of working memory regressed onto total WMH, controlling for age, sex, level of education, and TIV, in young/middle-aged adults (*N* = 86, mean age 37.41 ± 12.85) on the left and in older adults (*N* = 51, mean age 71.20 ± 6.26) on the right. *p* values < 0.05 are in bold

We first assessed the relationships between WMH and cognitive scores, adjusted for age, sex, level of education, and TIV (model A). Worse performance in executive functions was associated with total WMH and frontal WMH (Table 4, Fig. 2). Associations were also found between executive functions and parietal WMH (*β* = − 0.20, uncorrected *p* = 0.02), occipital WMH (*β* = − 0.22, uncorrected *p* = 0.01), and callosal WMH (*β* = − 0.20, uncorrected *p* = 0.02), but the associations did not survive Bonferroni correction for multiple comparisons (Bonferroni-corrected *p* = 0.09, 0.05, and 0.08, respectively). There was no interactive effect of the age group on the relationships between WMH and executive functions (*p* > 0.30 in all regions), suggesting that the association between WMH and executive functions was present across the entire adult lifespan, and not only in a specific age group. Composite scores of episodic memory, processing speed, and working memory were not significantly associated with WMH (Fig. 2, Additional file 1: Table S3), but there was a significant interaction between WMH and age group on working memory, suggesting a different effect of WMH on working memory in each group (*p* = 0.02). In the subgroup of young/middle-aged adults, total WMH were significantly associated with working memory (*β* = − 0.28, *p* = 0.02). No association was found in the subgroup of older adults (*β* = − 0.14, *p* = 0.36) (Fig. 3).

**Table 4:** Association between WMH, Aβ, and executive functions

		Total			Frontal			Parietal			Temporal			Occipital			Corpus callosum		
		*β*	*p*	*R*^2^	*β*	*p*	*R*^2^	*β*	*p*	*R*^2^	*β*	*p*	*R*^2^	*β*	*p*	*R*^2^	*β*	*p*	*R*^2^
*Model A*	**WMH**	− 0.23	**0.048**	0.30	− 0.25	**0.02**	0.31	− 0.20	0.09	0.29	− 0.06	1.00	0.27	− 0.22	0.05	0.30	− 0.20	0.08	0.29
*Model B*	**WMH**	− 0.32	**0.01**	0.30	− 0.33	**0.00**	0.31	− 0.28	**0.02**	0.29	− 0.15	0.62	0.25	− 0.32	**0.01**	0.31	− 0.26	**0.04**	0.28
	**Aβ**	0.06	1.00		0.05	1.00		0.07	1.00		0.10	1.00		0.09	1.00		0.06	1.00	
*Model C*	**WMH × Aβ**	− 0.07	1.00	0.30	0.61	1.00	0.31	− 0.15	1.00	0.29	− 0.14	1.00	0.24	− 0.70	1.00	0.31	0.45	1.00	0.28

Standardized betas (*β*), corrected *p* values, and adjusted *R*^2^ values are reported from regression models where composite scores of executive functions are regressed onto WMH (model A), WMH and Aβ (model B), and the interaction between WMH and Aβ (model C). All models are controlled for age, sex, level of education, and TIV. Significant *p* values (< 0.05) after applying Bonferroni correction (accounting for multiple testing across the 4 cognitive domains) are in bold. Analyses were performed with 136 participants in model A and with 109 participants in models B and C

*WMH* white matter hyperintensities, *Aβ* cortical β-amyloid

The association between total or regional WMH and executive functions found in the entire sample was still significant when adding Aβ load in the model, suggesting that WMH effect is independent from Aβ load. Aβ load was not associated with executive functions in that model (Table 4, model B). This result remained unchanged when Aβ was considered as a binary variable. Finally, there was no significant interaction between Aβ load and total or regional WMH on executive functions (Table 4, model C).

## Discussion

Our aim was to highlight the links between total and regional WMH, age, vascular risk factors, Aβ, and cognitive performance across the adult lifespan. We found that (i) WMH increased with age, in a steeper way in older age but already significantly in young and middle-aged adults; WMH also showed an age-dependent relationship with vascular risk factors; (ii) frontal, temporal, and occipital WMH were associated with higher Aβ, but this association was no longer significant when controlling for age and vascular risk factors; and (iii) higher total and frontal WMH were associated with lower performance in executive functions across the population including young and middle-aged adults, but not with lower performance in episodic memory and processing speed. Working memory was associated with WMH only in the subgroup of young/middle-aged adults. This effect of WMH was not driven by Aβ load, and there was no interaction between WMH and Aβ load on cognition.

The increase in WMH with age is classically described in older adults [43]. Given the presumed vascular origin of WMH, it is thought to reflect, at least partly, small vessel disease, which is an age-related disease [44]. While studies in older adults are numerous and constantly report increased WMH with age, fewer studies have assessed this question in younger participants, i.e., before 60 years old [3–7]. These studies found an age-related increase in WMH already in middle-aged adults, probably from the fifth or sixth decade [3, 4, 7]. An inflection point of 43 years old, where total WMH would start increasing with age, was recently reported [7]. However, this increase likely starts even earlier as WMH were found to be common already in the brains of healthy individuals aged 44–48 years, with an estimated prevalence of 50.9% [5]. Our findings support this hypothesis as they show an age-related increase in WMH even in young and middle-aged adults (and even in a subgroup of participants under 40 years old). Altogether, these findings show that WMH are not only accumulating in old ages but are also slightly increasing with age already in middle-aged and even in young adults.

These younger life periods are considered as critical for cerebrovascular health and might thus represent a strategic time window for preventive trials [12, 14, 15]. Previous studies classically report a link between WMH and vascular risk factors, and a recent report, including more than 8000 participants, confirmed the link between WMH and hypertension, tobacco smoking, and diabetes [4, 42, 45]. In the present study, increased WMH were associated with higher SBP, DBP, or HbA1C. This association was no longer significant when age and other covariates (model 2) were taken into account (except for SBP and parietal WMH), suggesting that age was the main driver of the link between WMH and vascular risk factors. This finding likely reflects the facts that participants were particularly healthy, our population was not enriched for vascular risk factors, and individuals with instable chronic disease or symptomatic cerebrovascular diseases were not included.

Higher frontal, temporal, and occipital WMH correlated to higher Aβ load. This association was not found with total WMH and with other regional WMH and was no longer significant in model 2, i.e., the multi-adjusted model including age and vascular risk factors as covariates. In the literature, results are also contrasted; while the majority of studies found no association between WMH and Aβ load [19], a recent study showed such an association in 424 participants aged 50 to 89 years old without dementia [20]. It is possible that this association is very subtle and only measurable in very large sample and/or in this specific age range.

We also found specific relationships between WMH and cognition and more specifically between increased total and frontal WMH and worse performance in executive functions. This relationship between WMH and cognition is consistent with many studies, altogether showing a small but robust effect of WMH on cognitive performance and especially on executive functions [2, 7, 46]. The specific relationship with executive functions, thought to be mainly supported by frontal areas, might reflect the fact that global and regional WMH disrupt long-distance white matter tracts notably involving connections with the frontal cortex [47, 48]. In line with this hypothesis, regional WMH, even non-frontal WMH, have been associated with lower glucose metabolism in frontal areas and executive functions in cognitively unimpaired older adults [49].

Most studies assessing the links between WMH and cognition focused on elderly populations. This question is rarely assessed in young and/or middle-aged adults, and results are contrasted [7–13]. While one previous study did not find any significant relationship between WMH and cognition in a group of cognitively unimpaired individuals including both young and middle-aged adults (i.e., from 20 to 60 years old) [8], more recent studies showed a negative association between total WMH and cognition in samples including both middle-aged and older participants (i.e., aged from 40 to 75 years old) [9–13]. We did not find the age group to affect the relationships between WMH and executive functions, suggesting that this effect is not limited to a specific age group but existed across the entire adult lifespan. There was an interaction between WMH and age group on working memory, suggesting a different effect of WMH on working memory between groups. Indeed, an association was found only in the subgroup of young/middle-aged adults, highlighting an early effect of WMH on working memory. The absence of significant association in the subgroup of older adults could be due to a lack of power related to the more limited size of the subgroup.

Pending replications, our findings thus suggest that WMH in adults, including young and middle-aged adults with very low total WMH (mean volume in the whole sample = 2.7 cm^3^), are associated with worse cognitive performance and should not be considered as fully silent (asymptomatic) lesions. These results highlight the importance to detect WMH in adults to propose interventions for secondary prevention at this early adulthood stage. We did not find significant associations between WMH and composite scores of episodic memory and processing speed, even if those associations have already been described [2, 7]. This might reflect the facts that these associations are more subtle and could only be detected in larger sample especially as our sample was very healthy and show limited variability, i.e., both low WMH severity and high cognitive performance.

Finally, we found that WMH effects on executive functions persisted when adding Aβ load in the models. This suggests that cortical Aβ, which is one of the main pathological landmark of Alzheimer’s disease, did not participate to the negative effect of WMH on executive functions in our sample. No interaction was found between WMH and Aβ suggesting that WMH and Aβ act rather independently as additive but not interactive processes. The additive or interactive relationship between cerebrovascular lesions, such as WMH, and Alzheimer’s pathology, including Aβ, is still debated. There are arguments supporting additive and independent effects of those lesions [50–52] while other studies argue that WMH and Aβ have synergistic associations with cognition [25, 26, 53]. The present study is not designed to address this specific question.

## Limitations

The main limitation is the observational and cross-sectional design of the study, preventing us from disentangling state effects from dynamic effects and from making causal interpretations. Another limitation is the use of automatic segmentation of WMH, which might be associated with increased risk of false positives or negatives compared to manual delineation. Also, we were specifically interested on WMH in the present study; for an overall comprehensive assessment of the effects of vascular lesions on cognition, other vascular lesions—such as cerebral lacunes or cortical microbleeds—should be considered. Finally, the measurement of Tau pathology, in vivo using PET with Tau radiotracers or with cerebrospinal fluid biomarkers, would allow assessing the interactions between both Alzheimer’s pathological landmarks and cerebrovascular pathology, even if recent studies failed to find an association between Tau pathology and WMH [20, 54].

The strengths and originality of this study rely on the presence of quantitative and homogeneous measurements of total and regional WMH on the one hand, and of Aβ on the other hand, combined with a detailed cognitive evaluation, in a sample of cognitively unimpaired individuals covering the entire adult lifespan, including young and middle-aged adults.

## Conclusion

This cross-sectional study found a significant effect of age on WMH across the adult lifespan, including in young and middle-aged adults. We highlighted associations between higher WMH and lower performance in executive functions, independently from Aβ, across the entire adult lifespan. These results suggest that WMH, even in young and middle-aged adults with low volumes, are clinically meaningful and should not be considered as fully silent lesions.

## Supplementary information

**Additional file 1: Table S1** Demographic, clinical and neuroimaging data in the subgroup of young/middle-aged adults and in the subgroup of older adults. **Figure S1.** Correlation matrices. **Figure S2** Relationships between regional WMH and age in the subgroup of young/middle-aged adults and in the subgroup of older adults, and interactive effects between age groups. **Table S2** Relationships between WMH, vascular risk factors and Aβ in the subgroup of young adults ≤40 years. **Figure S3** Total WMH as a function of age in adults younger than 40 years old. **Table S3** Associations between WMH and cognition in the whole sample.

## Data Availability

Data related to the current study are derived from the IMAP study. The datasets used and/or analyzed during the current study are available from the corresponding author on reasonable request.

## Abbreviations

Aβ: Cortical β-amyloid
DBP: Diastolic blood pressure
FDR: False discovery rate
HbA1C: Glycated hemoglobin
FLAIR: Fluid-attenuated inversion recovery
SUVR: Standardized uptake value ratio
SBP: Systolic blood pressure
TIV: Total intracranial volume
WMH: White matter hyperintensities
