# Supplementary material for: White matter hyperintensities across the adult lifespan: relation to age, Aβ load, and cognition
Source: Alzheimers Res Ther. 2020 Oct 8;12:127. doi: 10.1186/s13195-020-00669-4 (PMC7545576; doi:10.1186/s13195-020-00669-4)
Supplement: Supplementary file 1 — Additional file 1: Table S1 Demographic, clinical and neuroimaging data in the subgroup of young/middle-aged adults and in the subgroup of older adults. Figure S1. Correlation matrices. Figure S2 Relationships between regional WMH and age in the subgroup of young/middle-aged adults and in the subgroup of older adults, and interactive effects between age groups. Table S2 Relationships between WMH, vascular risk factors and Aβ in the subgroup of young adults ≤40 years. Figure S3 Total WMH as a function of age in adults younger than 40 years old. Table S3 Associations between WMH and cognition in the whole sample. [file 13195_2020_669_MOESM1_ESM.docx]

# Additional file 1

Table S1 Demographic, clinical and neuroimaging data in the subgroup of young/middle-aged adults and in the subgroup of older adults.

|  | Young/middle-aged adults | Older adults |
| --- | --- | --- |
| N | 86 | 51 |
| Age | 37.41 ± 12.85 [19 - 60] | 71.20 ± 6.26 [61 - 85] |
| Sex (% male) | 52 (N=45) | 47 (N=24) |
| Level of education (years) | 13.67 ± 2.80 [9 - 20] | 12.16 ± 3.84 [7 - 20] |
| SBP (mmHg) | 127.32 ± 14.68 [102 - 178] | 145.60 ± 20.96 [95 - 198] |
| DBP (mmHg) | 75.91 ± 9.44 [54 - 102] | 82.58 ± 12.38 [60 - 118] |
| HbA1C (%) | 5.30 ± 0.36 [4.50 - 6.80] | 5.73 ± 0.31 [5.20 - 6.60] |
|  |  |  |
| Episodic Memory | 0.16 ± 0.63 [-1.41 - 1.89] | -0.17 ± 0.72 [-2.11 - 1.22] |
| Processing Speed | 0.36 ± 0.60 [-1.17 - 1.77] | -0.62 ± 0.83 [-2.77 - 0.92] |
| Working Memory | 0.18 ± 0.86 [-1.60 - 2.58] | -0.30 ± 0.77 [-1.60 - 1.82] |
| Executive Functions | 0.16 ± 0.51 [-1.16 - 1.41] | -0.26 ± 0.79 [-2.66 - 1.13] |
|  |  |  |
| Total WMH (cm³) | 0.57 ± 0.72 [0 - 3.44] | 6.40 ± 6.87 [0.28 - 34.90] |
| Frontal WMH (cm³) | 0.10 ± 0.21 [0 - 1.01] | 2.26 ± 2.84 [0.04 - 14.92] |
| Parietal WMH (cm³) | 0.06 ± 0.14 [0 - 0.74] | 1.71 ± 2.22 [0 - 9.57] |
| Temporal WMH (cm³) | 0.05 ± 0.12 [0 - 0.76] | 0.81 ± 0.77 [0.01 - 3.11] |
| Occipital WMH (cm³) | 0.10 ± 0.14 [0 - 0.61] | 0.74 ± 0.89 [0 - 4.65] |
| Callosal WMH (cm³) | 0.07 ± 0.10 [0 - 0.53] | 0.78 ± 0.90 [0.02 - 4.09] |
| Aβ load (SUVR) | 1.15 ± 0.06 [0.99 - 1.26] | 1.20 ± 0.14 [0.97 - 1.64] |
| TIV (dm³) | 1.38 ± 0.12 [1.09 - 1.62] | 1.36 ± 0.14 [1.11 - 1.62] |

Numbers are expressed as mean ± standard deviation; the ranges are shown in brackets, or percentage (for the sex).

*SBP* Systolic Blood Pressure; *DBP* Diastolic Blood Pressure; *HbA1C* Glycated Hemoglobin; *WMH* White Matter Hyperintensities; *TIV* Total Intracranial Volume.

Figure S1. Correlation matrices

**
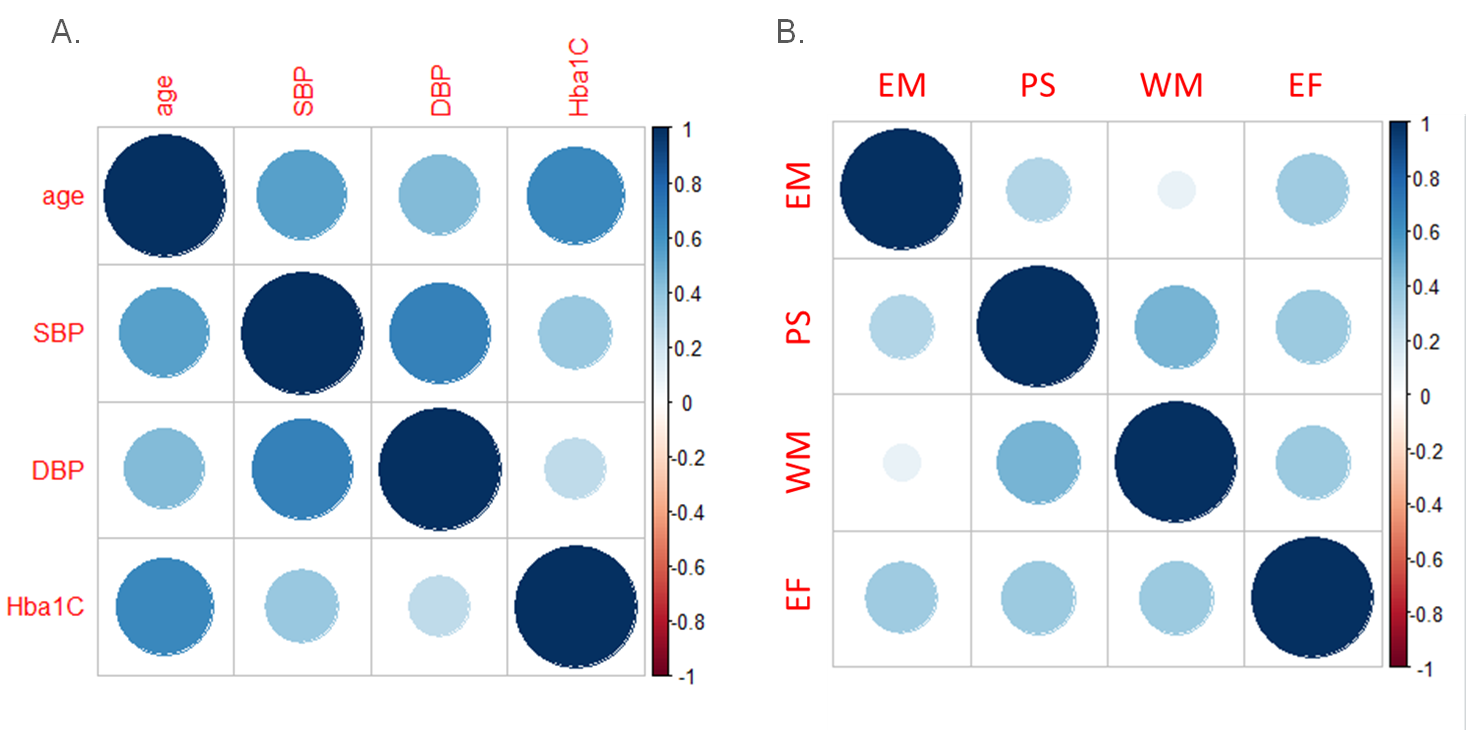
**

(A) Correlations between age, systolic blood pressure (SBP), diastolic blood pressure (DBP) and glycated hemoglobin (HbA1C). (B) Correlations between composite scores of episodic memory (EM), processing speed (PS), working memory (WM) and executive functions (EF).

Figure S2 Relationships between regional WMH and age in the subgroup of young/middle-aged adults and in the subgroup of older adults, and interactive effects between age groups.

**
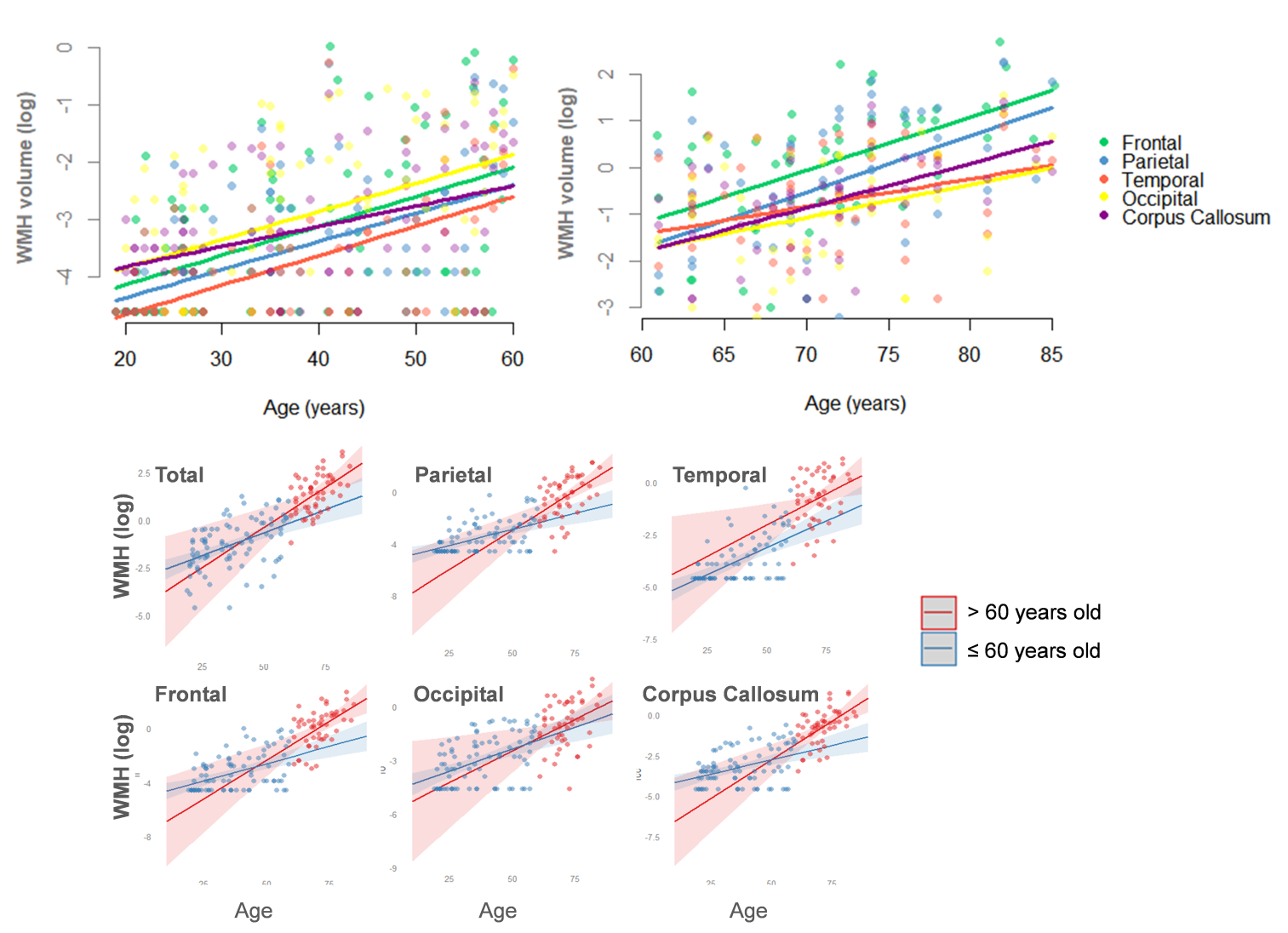
**

Plots represent the links between age and log-transformed regional WMH within the two age groups (on the top) and unadjusted interactive plots (on the bottom).

Table S2 Relationships between WMH, vascular risk factors and Aβ in the subgroup of young adults ≤ 40 years.

| ≤ 40 years old | Total WMH | | |
| --- | --- | --- | --- |
|  | *Model 1* | | |
|  | β | p | R² |
| Age | 0.32 | **0.01** | 0.27 |
| Sex | 0.00 | 0.99 | 0.17 |
| SBP | 0.05 | 0.70 | 0.17 |
| DBP | 0.14 | 0.29 | 0.19 |
| HbA1C | 0.11 | 0.41 | 0.17 |
| Aβ | 0.19 | 0.22 | 0.15 |

Standardized betas (β) and p-values are reported from regression models where total WMH (log-transformed) are regressed onto age, sex, SBP, DBP, HbA1C, Aβ controlling for TIV (model 1) in young adults (N = 50, mean age 27.86 ± 6.13). Adjusted R² values are indicated. Significant p-values (<0.05) are in bold.

Analyses were performed with 50 participants for age and sex, 49 participants for SBP and DBP, 48 participants for HbA1C and 38 participants for Aβ.

*SBP* Systolic Blood Pressure; *DBP* Diastolic Blood Pressure; *HbA1C* Glycated Hemoglobin; *Aβ* cortical β-Amyloid; *TIV* Total Intracranial Volume

Figure S3 Total WMH as a function of age in adults younger than 40 years old.


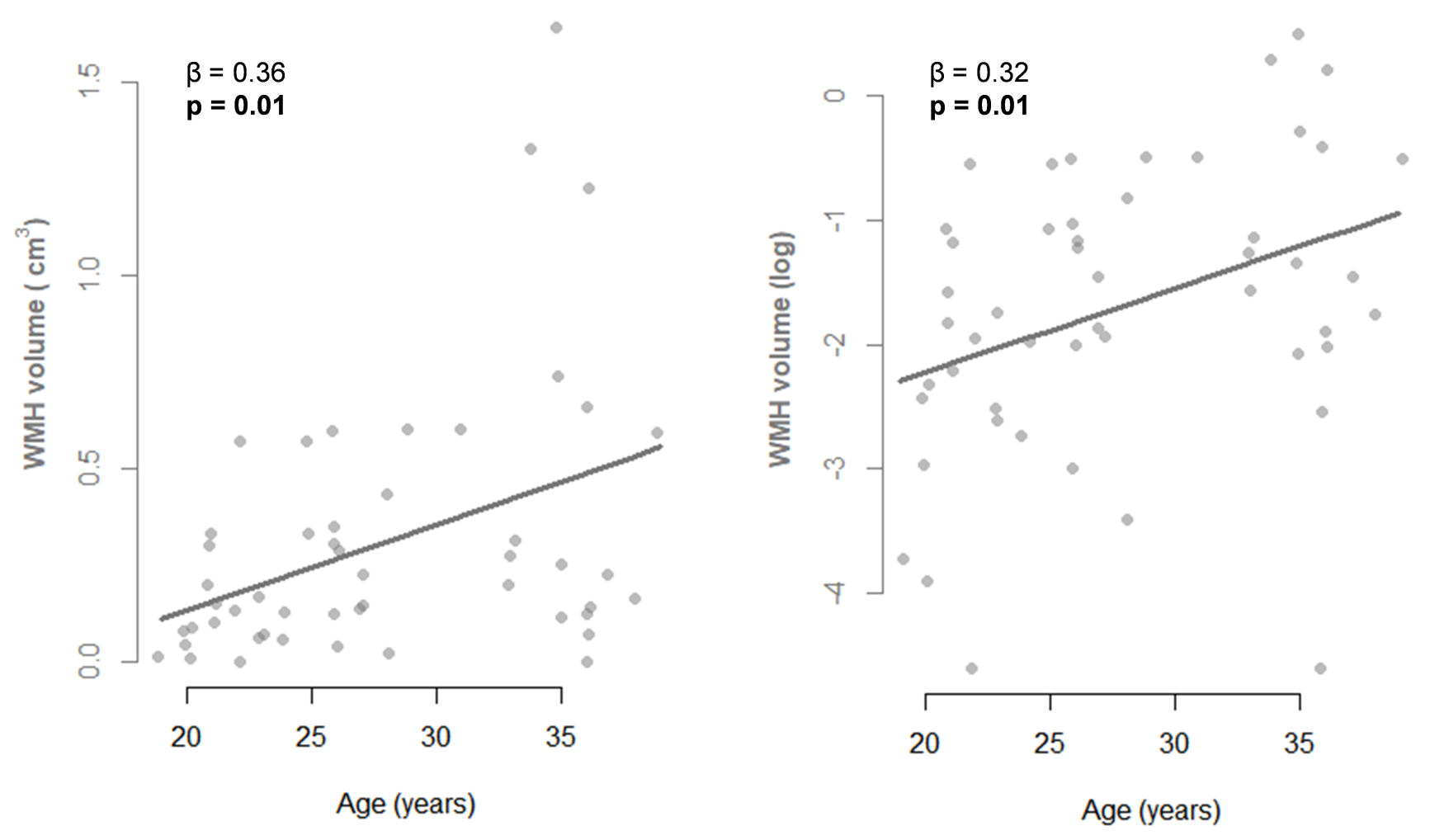


Plots represent the links between age and raw WMH on the left and the links between age and log-transformed WMH on the right.

P-values < 0.05 are in bold.

Table S3 Associations between WMH and cognition in the whole sample.

|  | Episodic Memory | | Processing Speed | | Working Memory | | Executive Functions | |
| --- | --- | --- | --- | --- | --- | --- | --- | --- |
|  | β | p | β | p | β | p | β | p |
| Total WMH | -0.16 | 0.12 | -0.07 | 0.40 | -0.08 | 0.39 | -0.23 | **0.01*** |
| Frontal WMH | -0.08 | 0.39 | -0.01 | 0.87 | -0.04 | 0.62 | -0.25 | **< 0.01*** |
| Parietal WMH | -0.18 | 0.07 | -0.08 | 0.28 | -0.06 | 0.55 | -0.20 | **0.02** |
| Temporal WMH | -0.13 | 0.20 | -0.09 | 0.25 | -0.12 | 0.22 | -0.06 | 0.53 |
| Occipital WMH | -0.17 | 0.08 | -0.10 | 0.22 | -0.08 | 0.36 | -0.22 | **0.01** |
| Callosal WMH | -0.10 | 0.31 | -0.05 | 0.49 | -0.12 | 0.20 | -0.20 | **0.02** |

Standardized betas (β) and p-values are reported from regression models where composite scores of episodic memory, processing speed, working memory and executive functions were regressed onto total and regional WMH, controlling for age, sex, level of education and TIV. Analyses were performed with 136 participants. Uncorrected p-values < 0.05 are in bold. * p < 0.05 after applying Bonferroni correction. *WMH* White Matter Hyperintensities
